# 1403. *Tuberculous sacroiliitis*: Clinical and Imaging Characteristics

**DOI:** 10.1093/ofid/ofab466.1595

**Published:** 2021-12-04

**Authors:** Fatma Hammami, Makram Koubaa, Amal Chakroun, Chakib Marrakchi, Fatma Smaoui, Mounir Ben Jemaa

**Affiliations:** Infectious Diseases Department, Hedi Chaker University Hospital, University of Sfax, Tunisia, Sfax, Sfax, Tunisia

## Abstract

**Background:**

Osteoarticular tuberculosis remains a common disease among which the spine is the most affected site. Less frequently, sacroiliac joint is involved. Its diagnosis is often delayed due to misleading and varied symptoms. The aim of this work was to study the clinical features and the contribution of imaging results in the diagnosis of tuberculous sacroiliitis.

**Methods:**

We conducted a retrospective study including all patients hospitalized in the infectious disease department for tuberculous sacroiliitis. The diagnosis was based on clinical, laboratory and radiological features.

**Results:**

In total, we encountered 12 women with a median age of 51 [39-63] years. Three patients had a family history of tuberculosis (25%). The median diagnostic delay was 155 [48-331] days. The revealing symptoms were lower back pain (75%) and hip pain (25%) associated with fever (83.3%) and weight loss (75%). Reduced mobility was noted in 3 cases (25%). Pulmonary tuberculosis and tuberculous spondylodiscitis were associated with tuberculous sacroiliitis in 5 cases (41.7%) and 4 cases (33.3%), respectively. Tuberculin skin test was positive in 6 cases (50%). Laboratory investigations revealed elevated C-reactive protein levels in 11 cases (91.6%) and accelerated erythrocyte sedimentation rates in 9 cases (75%). Needle biopsy of the sacroiliac joint (41.7%) and soft tissues abscess puncture (16.6%) were performed. Computed tomography scan revealed joint space widening (83.3%), peripheral joint erosions (83.3%) and osteolysis (58.3%). Soft tissue abscesses were noted in 66.7% of the cases. Magnetic resonance imaging was performed in 4 cases (33.3%). Sacroiliac joint was hypointense in T1-weighted images (75%), hyperintense in T2 weighted images (50%) and in STIR images (50%). Bone scintigraphy, performed in 5 cases, revealed hyperfixation of the sacroiliac area (100%). All patients received antitubercular therapy. Percutaneous abscess drainage was indicated in 4 cases (33.3%).

**Conclusion:**

Because of its deep localization, the diagnosis of tuberculous sacroiliitis is mainly based on imaging results associated with epidemiological, clinical and laboratory features. Antitubercular therapy initiated promptly leads to recovery.

**Disclosures:**

**All Authors**: No reported disclosures

